# Marek’s disease vaccines-induced differential expression of known and novel microRNAs in primary lymphoid organ bursae of White Leghorn

**DOI:** 10.1186/s13567-020-00746-4

**Published:** 2020-02-24

**Authors:** Lei Zhang, Chen Zhu, Mohammad Heidari, Kunzhe Dong, Shuang Chang, Qingmei Xie, Huanmin Zhang

**Affiliations:** 1 Avian Disease and Oncology Laboratory, USDA-ARS, East Lansing, MI 48823 USA; 2 Institute of Special Animal and Plant Sciences, Chinese Academy of Agricultural Sciences, Changchun, Jilin, 130112 China; 3 Michigan State University, East Lansing, MI 48824 USA; 4 Department of Pharmacology and Toxicology, Augusta University, Augusta, GA 30912 USA; 5 College of Veterinary Medicine, Shandong Agricultural University, Tai’an, Shandong 271018 China; 6 College of Animal Science, South China Agricultural University, Guangzhou, 510642 China

## Abstract

Marek’s disease (MD) is a contagious disease of domestic chickens caused by MD viruses. MD has been controlled primarily by vaccinations, yet sporadic outbreaks of MD take place worldwide. Commonly used MD vaccines include HVT, SB-1 and CVI988/Rispens and their efficacies are reportedly dependent of multiple factors including host genetics. Our previous studies showed protective efficacy of a MD vaccine can differ drastically from one chicken line to the next. Advanced understanding on the underlying genetic and epigenetic factors that modulate vaccine efficacy would greatly improve the strategy in design and development of more potent vaccines. Two highly inbred lines of White Leghorn were inoculated with HVT and CVI988/Rispens. Bursa samples were taken 26 days post-vaccination and subjected to small RNA sequencing analysis to profile microRNAs (miRNA). A total of 589 and 519 miRNAs was identified in one line, known as line 6_3_, 490 and 630 miRNAs were identified in the other, known as line 7_2_, in response to HVT or CVI988/Rispens inoculation, respectively. HVT and CVI988/Rispens induced mutually exclusive 4 and 13 differentially expressed (DE) miRNAs in line 6_3_ birds in contrast to a non-vaccinated group of the same line. HVT failed to induce any DE miRNA and CVI988/Rispens induced a single DE miRNA in line 7_2_ birds. Thousands of target genes for the DE miRNAs were predicted, which were enriched in a variety of gene ontology terms and pathways. This finding suggests the epigenetic factor, microRNA, is highly likely involved in modulating vaccine protective efficacy in chicken.

## Introduction

Marek’s disease (MD) is a proliferative disease of domestic chickens caused by Gallid alphaherpesvirus 2 (*Herpesviridae*), commonly known as MD virus (MDV). MD was first described by József Marek, a Hungarian veterinarian [1]. Clinical symptoms of MD vary among different lines of chickens and are dependent of exposure to different strains of MDV [2, 3], but commonly include polyneuritis, visceral lymphoma, acute transient paralysis, immunosuppression, brain edema, and acute rash [4, 5]. The morbidity and mortality could range from 11 [6] up to 100% [3]. Although MD has been well under control most of the time in most regions of the world by wide use of MD vaccines and improved management measures since the 1970s [7–9], sporadic outbreaks still occur from time to time all over the world. MD continues to pose a real threat and reportedly costs more than 2 billion U.S. dollars annually to the poultry industry [9, 10].

There are three commonly used MD vaccines commercially available up to date in most parts of the world. They are CVI988/Rispens, SB-1, and HVT, which were derived from MDV-1, MDV-2, and MDV-3 (also known as serotype 1, 2, and 3 MDV), respectively [11–13]. The SB-1 and HVT are naturally non-oncogenic. The CVI988/Rispens is a product of serial passaging in tissue culture and is considered an attenuated strain of live MDV-1. Like SB-1 and HVT, CVI988/Rispens is infectious but incapable of inducing lymphoid organ atrophy or tumor formation [14]. CVI988/Rispens and HVT have been used individually or in different combination of bivalent or trivalent format along with SB-1 on commercial farms worldwide [14]. Most users and researchers believe CVI988/Rispens is the most protective one of all commercially available vaccines against MD in chickens [15, 16], and HVT is no longer sufficiently protective or far less protective than CVI988/Rispens in preventing tumor formation against very virulent strains of MDV induction in chickens [17–20].

Protective efficacy of a vaccine is not only determined by the potency of the vaccine itself, but also host genetics of the recipients. By using *B*-congenic lines of experimental chickens [21] in trials of vaccination followed by MDV challenge, it was clearly shown that chicken major histocompatibility complex (*MHC*) *B*-haplotype poses significant influence on vaccinal immunity against MD [22]. This important finding was also subsequently demonstrated to hold true in commercial chickens [23]. Based on the data from those studies, chickens of B*2/B*2, B*13/B*13, B*15/B*15 and B*21/B*21 haplotypes gain the best protection against MD by MDV-1 vaccines like CVI988/Rispens; B*5/B*5 haplotype chickens are better protected by MDV-2 vaccines like SB-1; and in those genetic lines, B*2/B*2 and B*13/B*13 chickens were not better protected against MD by none of the MDV-1, MDV-2, and MDV-3 vaccines in contrast to other haplotypes of chickens.

In addition to *B*-haplotypes, non-*MHC* host genetic components also play a significant role modulating MD vaccine efficacy against MD [24]. The two highly inbred genetic lines of chickens, lines 6_3_ and 7_2_, maintained at USDA, Agricultural Research Service, Avian Disease and Oncology Laboratory (East Lansing, Michigan, USA), share a common B*2 haplotype, but line 6_3_ is relatively resistant and line 7_2_ is highly susceptible to MD [3]. Data from vaccination and challenge trials conducted in recent years showed that line 6_3_ chickens can convey better or equivalent protective efficacy in response to MDV-3 HVT vaccination than MDV-1 CVI988/Rispens. In a sharp contrast, the line 7_2_ birds respond to HVT very poorly and can convey some protection in response to CVI988/Rispens [25].

As early as in the 1980s, researchers realized that new routes in development of safer and more effective vaccines could be opened upon advances in molecular biology, genetics, and epigenetics [26, 27]. New evidences in the molecular, genomic and epigenomic levels in response to vaccination should highly likely lead to better understanding on protective efficacy of vaccines against infectious diseases including virus-induced cancers and offer new insights of host immune defenses as well as interaction between host immune system and vaccines. This study was designed to profile microRNAs (miRNA) in two genetically divergent lines of chicken post MD vaccine inoculation and to explore differentially expressed miRNAs in response to vaccination by CVI988/Rispens or HVT in contrast to non-vaccinated birds.

## Materials and methods

### Experimental animals

Day-old chicks were randomly sampled from two highly inbred lines of White Leghorns, known as the line 6_3_ and line 7_2_ chickens, maintained on the Avian Disease and Oncology Laboratory farm at East Lansing, Michigan, USA. Both lines are B*2 haplotype homozygous but drastically differ in genetic resistance to MD. Line 6_3_ is resistant and line 7_2_ is highly susceptible [21]. The two lines of chickens also drastically differ in response to MD vaccines [28].

### Challenge trial

The sampled 1-day old chicks from line 6_3_ and line 7_2_ were randomly divided into two groups per line. One group was inoculated with HVT intraperitoneally (IP) at a dose of 2000 plaque-forming units (PFU) each; and the other group was inoculated with CVI988/Rispens (IP) with the same dose. In addition, a control group was also included with the same sample size for each of the chicken lines under the same conditions in conjunction with a joint project simultaneously (to minimize the use of animals per experiment). The corresponding control group datasets were used in this study only to examine the differential expression of miRNAs in response to HVT and CVI988/Rispens inoculation. All chickens used in this study were housed in a BSL-2 experimental facility during the trial. Feed and water were supplied ad libitum. The chickens were observed daily throughout the entire duration of the experiment. This animal experiment was approved by USDA, Avian Disease and Oncology Laboratory Institutional Animal Care and Use Committee (IACUC). The IACUC guidelines established and approved by the ADOL IACUC (April 2005) and the Guide for the care and use of Laboratory Animals by Institute for Laboratory Animal Research (2011) were closely followed throughout the experiment.

### Total RNA extraction

Three chickens from each group were randomly euthanized at 26 days post-inoculation. Bursa samples were individually collected, and immediately placed into RNAlater solution (Qiagen, Valencia, CA, USA). The collected samples were stored at −20 °C until extractions of the total RNA samples. Total RNA samples were extracted with TRIzol reagent (Invitrogen, Carlsbad, CA, USA) following the manufacturer’s instructions.

### Small RNA sequencing

Total RNA samples were quantitatively and qualitatively checked with a NanoDrop 8000 Spectrophotometer (Thermo Fisher Scientific, Waltham, MA, USA) and an Agilent 2100 Bioanalyzer (Agilent Technologies, Santa Clara, CA, USA), respectively. Good quality RNA samples were chosen to construct standard cDNA libraries using Illumina TruSeq Small RNA Library Preparation kits following the manufacturer’s recommendations. Completed libraries were subjected to routine quality control (QC) checks and quantified using a combination of Qubit dsDNA HS and Agilent Bioanalyzer High Sensitivity DNA assays. The libraries were sequenced on an Illumina HiSeq 4000 sequencer using SBS (Sequencing by Synthesis) reagents. Base calling was accomplished by use of Illuima Real Time Analysis (RTA) v2.7.7 and the output of RTA was demultiplexed and then converted to FastQ format data with Ilunima Bcl2fastq v2.19.1 (Illumina, San Diego, CA, USA). The small RNA sequencing datasets supporting the results and conclusions of this article are available in the NCBI SRA repository [29]. The sequence datasets (accession numbers: SAMN11674924 to SAMN11674929) of the unvaccinated line 6_3_ and 7_2_ control groups used in the comparison analyses of miRNA differential expression were also deposited to NCBI SRA repository. The small RNA sequencing operations, including library preparation and preliminary reads quality control, were performed at the Research Technology Support Facility, Michigan State University (East Lansing, MI, USA).

### Small RNA_Seq reads data analyses

Small RNA_Seq reads data files that passed QC were analyzed one at a time with the miRDeep* software v3.8 [30] using the default parameters except the adapter sequence and the chicken genome build index files. The adapter sequence used in the analysis is TGG AAT TCT CGG GTG CCA AGG AAC TCC AGT CAC (Illumina); and the chicken genome build index (build_bwt_idx) files were constructed based on the chromosome information of the galGal 5.0 genome build. In addition, the “knownMiR.gff” file used in miRDeep* analysis of this study was the “gga.gff3” file at the miRbase download website [31, 32], which was constructed in accordance to galGal 5.0 assembly. Target genes of differentially expressed miRNAs were predicted using the built-in target gene prediction function in miRDeep*, which employees the most commonly used target gene prediction tool, TargetScan, in predicting the target genes of known and novel miRNAs [30, 33].

### Droplet digital™ PCR analysis

To validate the miRNA expressions derived from small RNA reads data, identified miRNAs were randomly elected to subject to droplet digital PCR (ddPCR) analysis to collect the absolute quantification reads of each miRNA from each of the total RNA samples of the four treatment groups (line 6_3_ HVT, line 6_3_ CVI988/Rispens, line 7_2_ HVT, and line 7_2_ CVI988/Rispens). Primers were designed for each of the selected miRNAs based on its mature sequence as described by Balcells et al. [34], which were used in the ddPCR (QX200™ ddPCR system; Bio-Rad Laboratories, Inc., Hercules, CA, USA) analysis. The cDNA samples used in ddPCR validation were reversely transcribed from individual RNA samples using the iScript™ RT Supermix Kit (Cat No. 170-8841) and following the manufacturer’s instructions (Bio-Rad). A ddPCR reaction of 25 μL in final volume was initially prepared per miRNA per biological sample containing 2 μL of cDNA, 12.5 μL of EvaGreen Supermix (Cat No. 1864034), 0.5 μL of each forward and reverse primers (200 nM; synthesized by Eurofins Genomics, Huntsville, AL), and 9.5 μL of nuclease-free water. Of which, 20 μL were loaded into one of 8 sample channels of a DG8™ cartridge (Cat No. 1864008, Bio-Rad). Each oil well was loaded with 70 μL of droplet generating oil (Cat No. 1864006, Bio-Rad). The loaded DG8™ cartridges were placed on a QX200™ droplet generator (Bio-Rad) to generate the digital droplets. Forty μL of the generated droplet emulsion for each sample were transferred to a well in a 96-well PCR plate followed by polymerase chain reaction with EvaGreen on a C1000™ Thermal Cycler (Bio-Rad). The cycling conditions were 95 °C for 5 min, followed by 40 cycles of 95 °C for 15 s, 58 °C for 60 s, and a final extension step of 98 °C for 10 min. The droplets post PCR were read well by well on a QX200™ droplet reader (Bio-Rad). PCR-positive and PCR-negative droplets in each of the wells were counted and analyzed with the QuantaSoft™ Software (Version 1.7, Bio-Rad).

### Differentially expressed miRNA identification and GO terms enrichment analysis

The number of reads per miRNA for each biological sample were counted using HTSeq [35]. In each of the pairwise comparisons (between MD vaccine inoculated group and the control group for each chicken line, between the two MD vaccinated groups for each chicken line, and between the two chicken lines for each vaccinated group), differentially expressed miRNAs were identified by use of a custom R script encompassing the DESeq R package (2.1.0). A filter criterion of *FDR* < 0.05 and FC > 2 was enforced. For some of the differentially expressed miRNAs that ended up with a zero-statistic estimate for a normalized average TPM (baseMeanA or baseMeanB) in a contrast, an arbitrary small value of 1 was assigned to substitute the zero in order to computer a numeric fold change, and then a log_2_ fold change value for easier comprehension of the estimates. To better understand the functional involvements of the identified miRNAs differentially expressed in response to the vaccination, predicted target gene lists of differentially expressed miRNAs for each of the contrasts were subjected to GO terms and pathway analysis using the g:Proflier [36] online tools with the following options: Organism: Gallus gallus; Statistical domain scope: All known genes; Significance threshold: Bonferroni correction; User threshold: 0.01 [37].

## Results

### Small RNA sequencing

Small RNA sequencing generated an average of 35.7 million PF reads (the number of clusters that passed Illumina’s “Chastity filter”) per biological sample, with a range of 23 to 50.8 million PF reads for all 12 bursal samples of the lines 6_3_ and 7_2_ chickens inoculated with either HVT or CVI988/Rispens. The raw sequence datasets (accession numbers: SAMN11675491 - SMAN11675502) are available at the Sequence Read Archive (SRA) under the National Centre for Biotechnology Information (NCBI) website with an assigned BioProject SRA accession number: PRJNA543526 [38].

### MicroRNA profiles

**Figure 1:**
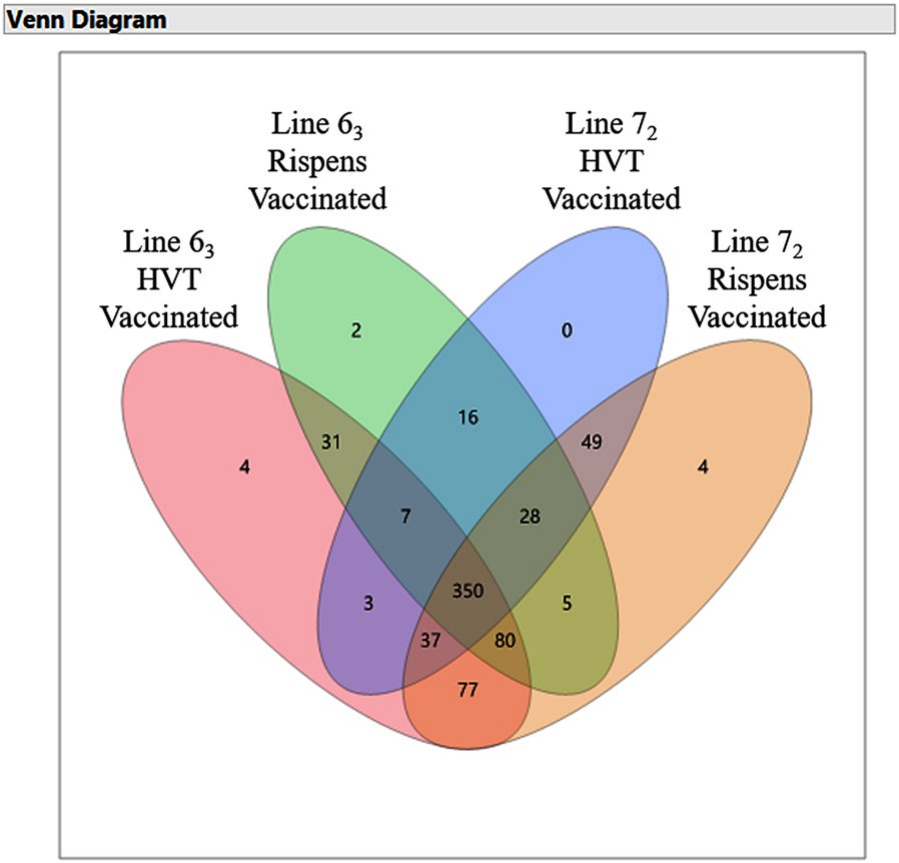
**A venn diagram depicting numbers of identified miRNAs.** The numbers of identified miRNAs exclusively or commonly expressed in one of or between the vaccinated treatment groups of the line 6_3_ and 7_2_ birds are graphically illustrated.

A total of 693 miRNAs was identified in this study, which were mapped to 31 pairs of chromosomes (gga chr1-28, chr32-33, chrW and Z), and was identified in no fewer than three biosamples with reads counts of five and above. Four hundred and ninety-five out of the 693 miRNAs were novel miRNAs (see Additional file 1 for a complete list of the identified miRNAs), which have been deposited to miRbase Registry for processing in validation and finalizing name assignment. The total numbers of identified miRNAs were 589 and 519 in bursae of the line 6_3_ birds, and 490 and 630 in bursae of the line 7_2_ birds inoculated with HVT or CVI988/Rispens, respectively (Table 1). The numbers of exclusively identified miRNAs and the numbers of identified miRNAs in common between vaccine treatment groups of the line 6_3_ and 7_2_ birds are depicted in a Venn diagram (Figure 1). The details of the identified miRNAs, including miRNA name (miRNA_ID), average normalized transcripts per million (Mean (TPM)), mature loci, chromosome (chr), hairpin and mature sequences, are given in Additional files 2, 3, 4, 5.

**Table 1:** **Numbers of microRNAs identified in bursae of line 6**_**3**_**and 7**_**2**_**chickens 26-days post HVT or CVI988/Rispens inoculation after hatch**

Chicken	Vaccine	Total number	Known miRNA	Novel miRNA
Line 6_3_	HVT	589	195	394
	CVI988/Rispens	519	187	332
Line 7_2_	HVT	490	188	302
	CVI988/Rispens	630	192	438

### Differentially expressed microRNAs in response to HVT vaccination

Four out of the 589 miRNAs identified in bursae of the line 6_3_ birds were differentially expressed in response to HVT vaccination in contrast to an unvaccinated group of line 6_3_ birds (1.33E−6 < *p* < 2.58E−4, 9.62E−4 < *FDR* < 4.68E−2). Two of the miRNAs were significantly upregulated (log_2_ Fold Change: 3.27 and 7.63), and the other two were significantly downregulated (log_2_ FC: −8.68 and −6.09). None of the identified miRNAs in bursae of the line 7_2_ birds was differentially expressed in response to HVT vaccination in contrast to its counterpart of an unvaccinated group (Table 2).

**Table 2:** **HVT and CVI988/Rispens-induced differentially expressed microRNAs in bursae within each line (6**_**3**_**and 7**_**2**_**) of chickens 26-days post HVT or CVI988/Rispens inoculation after hatch**

miRNA_ID	HVT-induced			Rispens-induced						Rispens/HVT induced					
	Line_6_3_			Line_6_3_			Line_7_2_			Line_6_3_			Line_7_2_		
	log_2_ FC	*p* value	*FDR*	log_2_ FC	*p* value	*FDR*	log_2_ FC	*p* value	*FDR*	log_2_ FC	*p* value	*FDR*	log_2_ FC	*p* value	*FDR*
novelMiR_203_4	−8.68	1.33E−06	9.62E−04							9.36	9.72E−27	1.04E−24			
novelMiR_396	−6.09	2.58E−04	4.68E−02							6.05	1.40E−05	5.25E−04			
novelMiR_483	3.27	1.35E−04	3.26E−02							−4.13	3.84E−07	1.92E−05			
novelMiR_909	7.63	1.20E−05	4.34E−03							−7.67	3.48E−06	1.54E−04			
gga-mir-205b				3.49	1.06E−04	6.39E−03									
novelMiR_1232				3.14	7.65E−04	4.27E−02				3.63	4.76E−06	1.99E−04			
novelMiR_1241				3.90	5.01E−06	3.49E−04				4.39	1.03E−09	6.46E−08			
novelMiR_1251				10.77	1.60E−134	1.16E−131	−10.01	4.52E−05	3.43E−02	11.26	3.05E−192	2.29E−189	−11.45	1.80E−07	1.37E−04
novelMiR_1279_1				5.44	2.22E−15	2.69E−13				5.94	1.64E−25	1.37E−23			
novelMiR_1279_2				5.44	2.22E−15	2.69E−13				5.94	1.64E−25	1.37E−23			
novelMiR_145_1				3.12	1.37E−27	3.31E−25				4.02	9.52E−61	1.79E−58			
novelMiR_145_2				3.12	1.32E−27	3.31E−25				4.02	9.00E−61	1.79E−58			
novelMiR_215				−4.69	2.14E−10	1.72E−08				−5.10	4.76E−13	3.25E−11	3.21	1.70E−04	4.29E−02
novelMiR_459				5.24	5.29E−06	3.49E−04				4.63	3.15E−08	1.82E−06			
novelMiR_488				3.06	3.05E−11	2.76E−09				6.79	4.75E−42	7.14E−40			
novelMiR_5				3.52	5.45E−19	9.88E−17				4.69	7.49E−28	9.38E−26			
novelMiR_52				3.21	3.74E−12	3.88E−10				4.11	2.74E−16	2.06E−14			
novelMiR_100_1										3.35	4.03E−04	1.08E−02			
novelMiR_100_2										3.35	4.03E−04	1.08E−02			
novelMiR_1304										2.72	1.73E−03	4.49E−02			
novelMiR_451										4.02	1.20E−07	6.43E−06			
novelMiR_476										3.53	3.44E−04	9.94E−03			
novelMiR_507_1										3.41	4.21E−05	1.32E−03			
novelMiR_507_2										3.37	2.80E−05	1.00E−03			
novelMiR_507_3										3.41	4.21E−05	1.32E−03			
novelMiR_507_4										3.41	4.21E−05	1.32E−03			
novelMiR_60										−3.75	1.32E−05	5.22E−04			
novelMiR_97_1										1.51	2.20E−06	1.03E−04			
novelMiR_97_2										9.05	1.15E−93	4.33E−91			

### Differentially expressed microRNAs in response to CVI988/Rispens vaccination

One of the novel miRNAs, novelMiR_215, was significantly downregulated (*p* = 2.14E−10, *FDR* = 1.72E−08) with a log_2_ FC = −4.69, and 12 miRNAs were significantly upregulated 1.60E−134 < *p* < 7.65E−4, 1.16E−131 < *FDR* < 4.27E−2) with a range of log_2_ FC from 3.06 to 10.77 in bursae of line 6_3_ birds in response to CVI988/Rispens vaccination. In bursae of line 7_2_ birds, CVI988/Rispens induced a single novel miRNA, novelMiR_1251, with significantly downregulated expression (log_2_ FC = −10.01, *p* < 4.53E−5, *FDR* = 3.43E−2) in contrast to line 7_2_ unvaccinated group (Table 2).

### Differentially expressed microRNAs in response to CVI988/Rispens in contrast to HVT vaccination within each of the chicken lines

**Figure 2:**
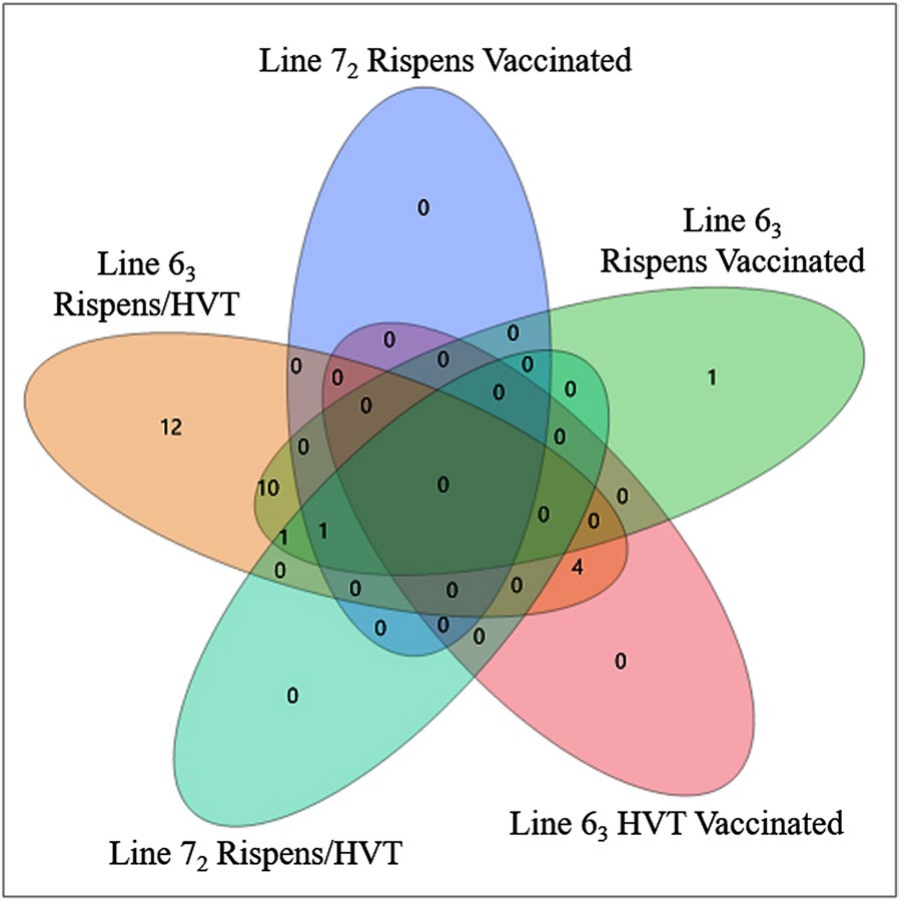
**A venn diagram graphically illustrating the numbers of differentially expressed miRNAs.** The numbers of differentially expressed miRNAs exclusively in one or commonly between vaccine treatment groups of a chicken line and between the lines as well as the comparisons between vaccine treatments within each of the lines are depicted.

Twenty-eight identified miRNAs were differentially expressed in bursae of the line 6_3_ birds in response to CVI988/Rispens inoculation in contrast to HVT inoculation. Four of the miRNAs were significantly downregulated with log_2_ FC ranged from −7.67 to −3.75 (4.76E−13 < *p* < 1.32E−5; 3.25E−11 < *FDR* < 5.22E−4). The other 24 miRNAs were significantly upregulated. The log_2_ FC ranged from 1.51 up to 11.26 (3.05E−192 < *p* < 1.73E−3; 2.29E−189 < *FDR* < 4.49E−2). In contrast to HVT inoculation, CVI988/Rispens-induced a single novel microRNA, novelMiR_1251, with significantly downregulated expression (log_2_ FC = −11.45; *p* < 1.80E−7 and *FDR* < 1.37E−4) and another novel microRNA, novelMiR_215, with significantly upregulated expression (log_2_ FC = 3.21; *p* < 1.70E−4 and *FDR* < 4.29E−2) in line 7_2_ birds (Table 2). The numbers of differentially expressed miRNAs in response to HVT or CVI988/Rispens inoculation and between the CVI988/Rispens and HVT vaccination within each of the two chicken lines are graphically illustrated in a Venn diagram (Figure 2).

### Differentially expressed microRNAs in response to HVT or CVI988/Rispens vaccination between line 6_3_ and line 7_2_ birds

There were 34 identified miRNAs (8 known-miRNAs and 26 novel miRNAs) differentially expressed in bursae between the line 6_3_ and 7_2_ birds 26 days post HVT inoculation. Eight of those miRNAs were significantly upregulated (log_2_ FC: 3.49 to 7.64; 3.66E−6 < *p* < 5.57E−4; 1.37E−4 < *FDR* < 1.31E−2) and 26 were downregulated (log_2_ FC: −11.45 to −1.24; 6.58E−47 < *p* < 1.89E−3; 5.09E−44 < *FDR* < 4.06E−2) in line 6_3_ birds in contrast to line 7_2_ in response to HVT inoculation. Thirty identified miRNAs (6 known-miRNAs and 24 novel miRNAs) were differentially expressed between line 6_3_ and 7_2_ birds post CVI988/Rispens inoculation. Twenty-four were significantly upregulated (log_2_ FC: 1.45 to 11.29; 5.67E−101 < *p* < 1.97E−3; 4.43E−98 < *FDR* < 4.80E−2), and the other 6 were downregulated (log_2_ FC: −6.71 to  −1.69; 4.31E−21 < *p* < 1.59E−3; 1.12E−18 < *FDR* < 4.01E−2) in response to CVI988/Rispens inoculation in line 6_3_ birds in contrast to line 7_2_ (Table 3).

**Table 3:** **Differentially expressed microRNAs in bursae between the line 6**_**3**_**and the line 7**_**2**_**birds 26-days post HVT or CVI988/Rispens inoculation after hatch**

miRNA_ID	Line_6_3_/Line_7_2_					
	HVT-induced			Rispens-induced		
	log_2_ FC	*p* value	*FDR*	log_2_ FC	*p* value	*FDR*
gga-mir-1684a	−4.99	4.21E−13	1.09E−10	−6.71	8.25E−10	7.16E−08
gga-mir-193b	−1.95	9.35E−06	4.30E−04	−1.69	1.27E−03	3.29E−02
gga-mir-31	−1.70	2.61E−04	7.21E−03			
gga-mir-499	−1.43	9.45E−06	4.30E−04			
gga-mir-6586	3.53	1.74E−04	5.17E−03	3.29	3.42E−04	1.07E−02
gga-mir-9-1	−1.24	3.97E−09	3.41E−07			
gga-mir-9-1*	−1.24	3.97E−09	3.41E−07			
gga-mir-9-2	−1.24	3.97E−09	3.41E−07			
novelMiR_102	3.83	2.23E−05	9.09E−04	3.64	3.46E−05	1.69E−03
novelMiR_117	−1.34	6.69E−05	2.16E−03			
novelMiR_1232	−3.24	1.12E−03	2.48E−02	3.67	3.39E−05	1.69E−03
novelMiR_1241	−4.02	6.36E−06	3.28E−04			
novelMiR_1251	−11.45	8.82E−08	5.69E−06	11.29	5.67E−101	4.43E−98
novelMiR_1279_1	−5.26	2.57E−05	9.47E−04	4.03	2.95E−07	1.77E−05
novelMiR_1279_2	−5.26	2.57E−05	9.47E−04	4.03	2.95E−07	1.77E−05
novelMiR_129_1	−1.24	3.97E−09	3.41E−07			
novelMiR_129_2	−1.24	3.97E−09	3.41E−07			
novelMiR_130	3.49	2.26E−04	6.48E−03			
novelMiR_1512_1	−4.46	3.97E−09	3.41E−07			
novelMiR_1565	−5.95	4.94E−24	1.91E−21	−6.35	4.31E−21	1.12E−18
novelMiR_1616	−2.96	1.02E−03	2.32E−02			
novelMiR_203_4	−8.05	3.49E−04	9.31E−03			
novelMiR_277	−2.81	1.89E−03	4.06E−02			
novelMiR_329	3.96	4.81E−04	1.16E−02			
novelMiR_369	−3.54	3.28E−05	1.10E−03			
novelMiR_396	−7.10	6.58E−47	5.09E−44			
novelMiR_434	4.13	3.85E−04	9.93E−03			
novelMiR_443	3.88	5.57E−04	1.31E−02			
novelMiR_451	−3.61	1.87E−05	8.05E−04			
novelMiR_459	−4.81	4.00E−04	9.99E−03	3.62	1.98E−04	6.72E−03
novelMiR_464	−4.14	2.78E−05	9.77E−04	−4.17	3.29E−06	1.83E−04
novelMiR_483	4.10	2.31E−06	1.37E−04			
novelMiR_488	−7.15	3.70E−08	2.60E−06	4.02	6.46E−10	6.31E−08
novelMiR_909	7.64	3.66E−06	2.03E−04			
gga-mir-1434				2.42	1.25E−03	3.29E−02
gga-mir-1655				2.94	1.97E−03	4.80E−02
gga-mir-1798				3.10	1.02E−03	2.84E−02
novelMiR_145_1				2.86	7.47E−14	9.72E−12
novelMiR_145_2				2.86	7.33E−14	9.72E−12
novelMiR_215				−5.71	4.45E−15	8.68E−13
novelMiR_268				2.41	5.20E−04	1.51E−02
novelMiR_474				11.06	2.34E−08	1.66E−06
novelMiR_475				8.01	1.29E−04	5.05E−03
novelMiR_5				2.85	1.17E−13	1.31E−11
novelMiR_507_1				3.46	1.04E−04	4.27E−03
novelMiR_507_2				3.42	1.39E−04	5.16E−03
novelMiR_507_3				3.46	1.04E−04	4.27E−03
novelMiR_507_4				3.46	1.04E−04	4.27E−03
novelMiR_52				2.80	3.01E−09	2.35E−07
novelMiR_60				−3.19	1.59E−03	4.01E−02
novelMiR_97_1				1.45	2.64E−04	8.58E−03
novelMiR_97_2				9.08	1.97E−66	7.69E−64

### ddPCR validation of miRNA expression determined by small RNA_Seq

**Figure 3:**
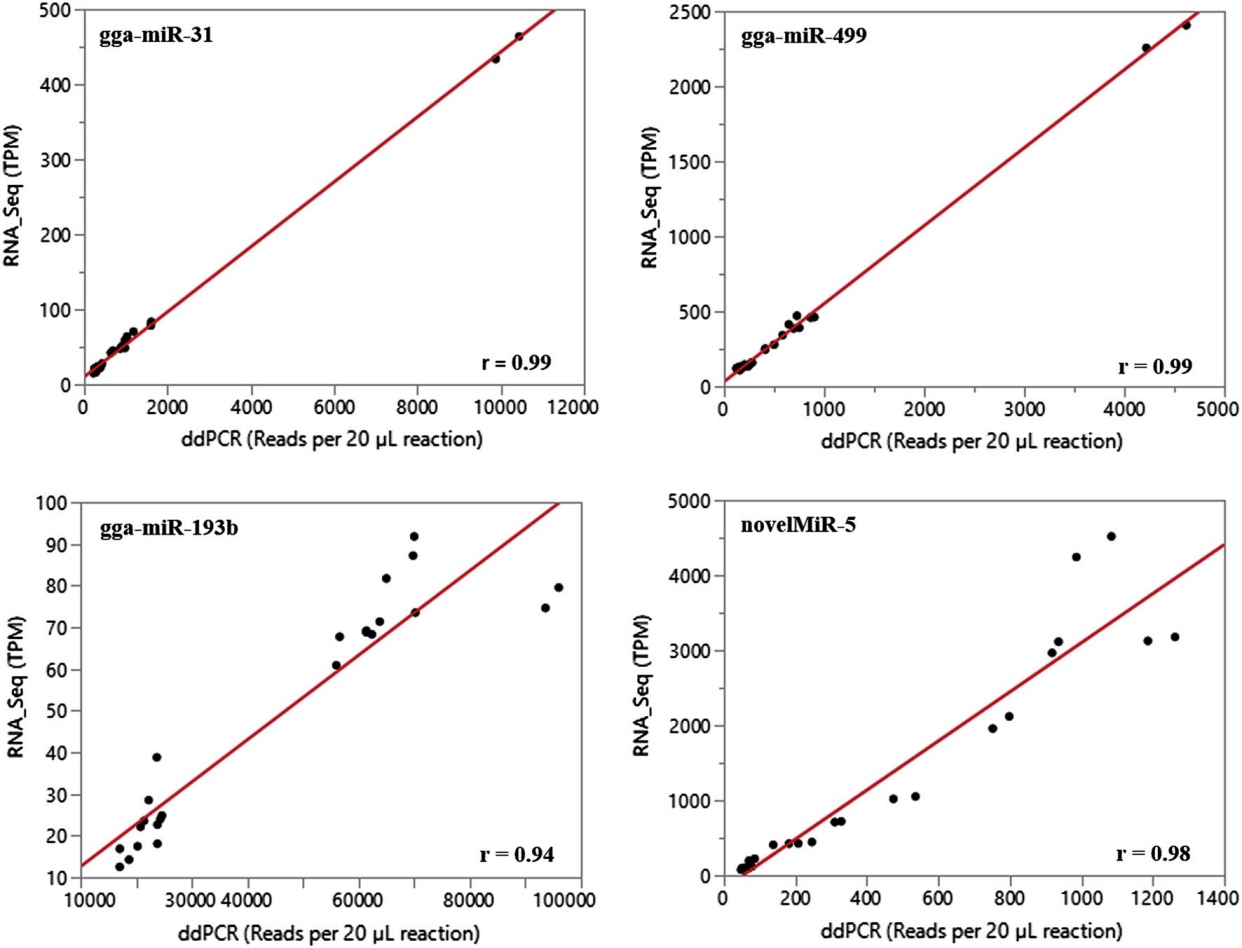
**Bivariate plots illustrating the relationship between small RNA_Seq data and droplet digital PCR data.** The normalized expression of small RNA_Seq data, transcripts per million, (TPM), and droplet digital PCR (ddPCR) data were analyzed by fitting a bivariate model. The bivariate plots for four of the validated miRNAs are given graphically showing the two sets of expression data. The correlation coefficients between the small RNA_Seq and the ddPCR data for these four miRNAs ranged from r = 0.94 (gga-miR-193b) to r = 0.99 (both gga-miR-31 and gga-miR-499), which provided highly positive support to the estimates of microRNA expression derived from the small RNA_Seq data.

**Figure 4:**
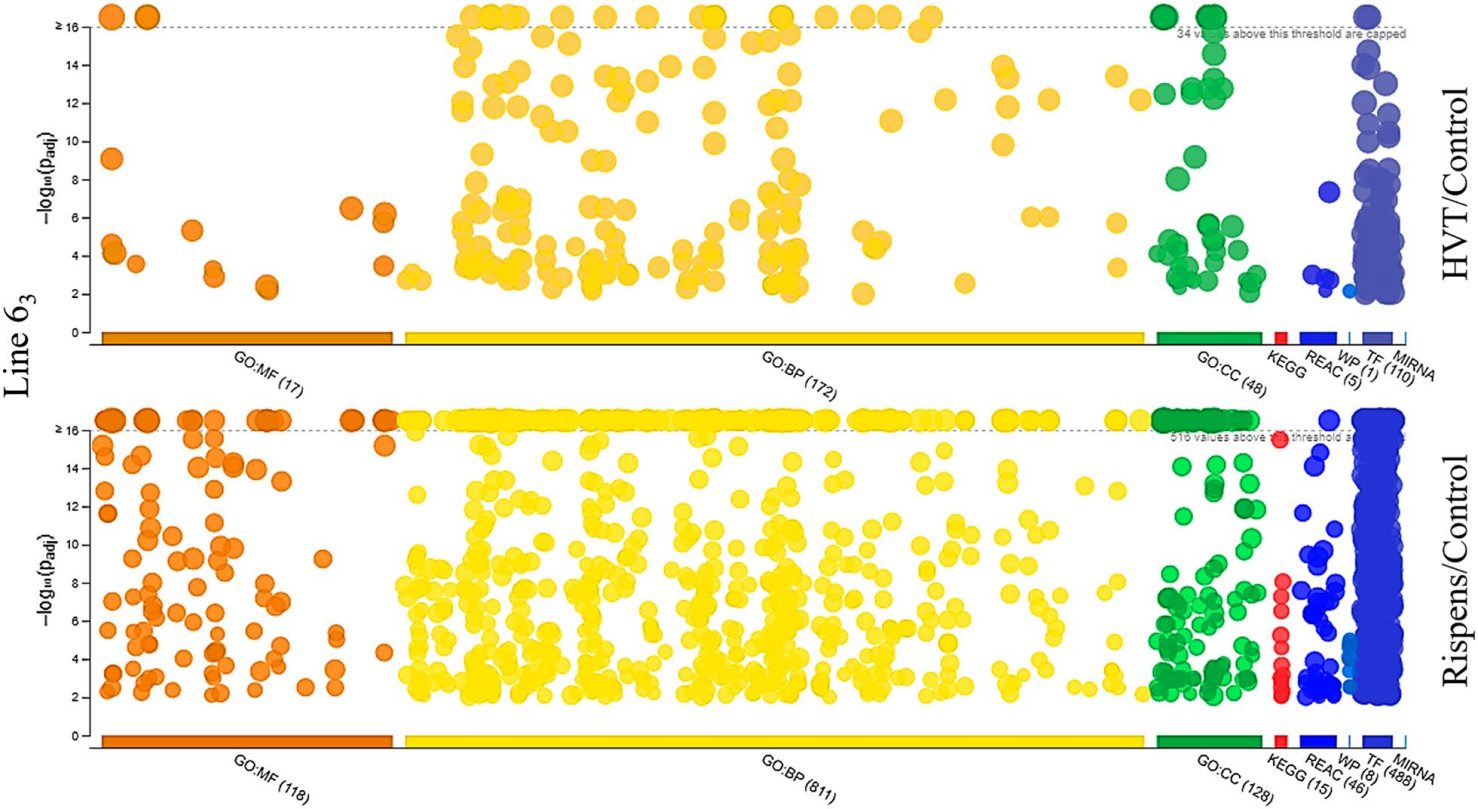
**Manhattan plots illustrating GO term enrichments of target genes.** The target genes of differentially expressed microRNAs of the line 6_3_ birds were analyzed by g:Profiler and the enrichment in GO terms (MF: molecular function; BP: biological process; CC: cellular component) and KEGG pathways across Reactome pathways (REAC), WiKi-Pathways (WP), transcription factor (TF), and microRNA target base (MIRNA) were graphically depicted in two plots. The plots depicted the enrichments of target genes for differentially expressed microRNAs of the line 6_3_ birds in response to HVT (top) and CVI988/Rispens (bottom) vaccination, respectively. A clearly visible difference in enrichments between the two treatment groups (HVT and CVI988/Rispens vaccination) is also demonstrated.

To validate the miRNA expression determined by small RNA_Seq analysis, ten of the identified miRNAs were selected to subject to absolute quantification of the expression by ddPCR. The selected miRNAs and the designed ddPCR primers are listed in Table 4. The summary statistics including correlation coefficients between the normalized small RNA_Seq reads and the ddPCR absolute quantification counts are given in Table 5. The correlation coefficients ranged from r = 0.63 (gga-miR-140) up to r = 0.99 (both gga-miR-31 and gga-miR-499) with a single *p* value smaller than 0.0001 (Table 5). Manhattan bivariate plots depicting the relationship between normalized expression of small RNA_Seq data (TPM) and the ddPCR absolute quantification reads for four of the selected miRNAs are given in Figure 3, which visually illustrates the validation. Taking all together, Table 5 and Figure 4 provided experimental evidence that highly positively supports the microRNA expression estimates derived from the small RNA_Seq data of this study.

**Table 4:** **Primers used in ddPCR to validate the expressions of a small subgroup of identified miRNAs, which were determined by small RNA_Seq analysis**

miRNA	Sequence	Forward primer (5′ → 3′)	Reverse primer (5′ → 3′)
gga-miR-31	aggcaagatgttggcatagctg	gcagaggcaagatgttggcat	caggtccagtttttttttttttttcagcta
gga-miR-193b	aactggcccacaaagtcccgct	cgcagaactggcccacaaag	gtccagtttttttttttttttagcgggact
gga-mir-140	accacagggtagaaccacggac	cagaccacagggtagaacca	aggtccagtttttttttttttttgtccgt
gga-mir-142	cataaagtagaaagcactact	cagcgcagcataaagtagaaagca	gcaggtccagtttttttttttttttagtagt
gga-miR-499	ttaagacttgtagtgatgttt	agcgcagttaagacttgtagtgat	agcaggtccagtttttttttttttttaaacat
gga-mir-153	ttgcatagtcacaaaagtgatc	gcgcagttgcatagtcacaaaag	ccaggtccagtttttttttttttttgatca
gga-mir-1677	ttgacttcagtaggagcaggatt	gcagttgacttcagtaggagca	gcaggtccagtttttttttttttttaatcct
gga-mir-1769	agtgtgaaatctgcctgaaagt	gcagagtgtgaaatctgcctga	gcaggtccagtttttttttttttttactttc
novelMiR_142	agccggggatgatttctgcct	cgcagagccggggatgatt	aggtccagtttttttttttttttaggcagaa
novelMiR-5	gtagtcgtggccgagtggttaag	ggtagtcgtggccgagtg	gcaggtccagtttttttttttttttcttaac

**Table 5:** **Summary statistics of ddPCR absolute counts and RNA_Seq reads for validation of miRNA expression data detected by small RNA sequencing**

miRNA	No. observations	r	*p* value
gga-miR-31	24	0.99	0.0001
gga-miR-193b	24	0.94	0.0001
gg-miR-140	24	0.63	0.001
gga-miR-142	24	0.87	0.0001
gga-miR-499	24	0.99	0.0001
gga-miR-153	24	0.85	0.0001
gga-miR-1677	24	0.77	0.0001
gga-miR-1769	24	0.98	0.0001
novelMiR-142	24	0.79	0.0001
nvelMiR-5	24	0.98	0.0001

### Predicted target genes of differentially expressed miRNAs

Varied numbers (12 up to 6153) of target genes were predicted for the differentially expressed miRNAs per contrast for all contrast groups except one, the line 7_2_ HVT treatment group over its counterpart of control group, which resulted in no differentially expressed miRNA. The numbers of differentially expressed miRNAs, predicted target genes, involved gene ontology (GO) terms and KEGG pathways are given in Table 6 by contrast group.

**Table 6:** Identified numbers of differentially expressed miRNAs, predicted target genes, enriched in number of GO terms and KEGG pathways per contrast group

Contrast	Differentially expressed miRNAs	Predicted target genes	Number of GO terms	Number of KEGG pathways
Line 6_3_ HVT/control	4	463	237	0
Line 6_3_ rispens/control	13	2928	1057	15
Line 7_2_ HVT/control	0	0	0	0
Line 7_2_ rispens/control	1	12	4	1
Line 6_3_ rispens/HVT	28	3677	1255	24
Line 7_2_ rispens/HVT	2	176	113	0
Line 6_3_ HVT/Line 7_2_ HVT	34	6153	1999	46
Line 6_3_ rispens/line 7_2_ rispens	30	5277	1577	32

### Target-gene-set enrichment in GO terms and pathways for differentially expressed microRNAs in response to HVT or CVI988/Riepsnes vaccination of line 6_3_ and 7_2_ birds

Target genes of differentially expressed miRNAs in response to HVT or CVI988/Rispens in line 6_3_ birds were highly enriched in a variety of GO terms and pathways. In contrast, HVT failed to induce any differentially expressed miRNA and CVI988/Rispens induced a single differentially expressed miRNA in line 7_2_ birds, which reportedly targets 12 functional genes. Therefore, there was only a very limited number of GO terms and pathways in which those target genes were enriched.

The target genes of HVT-induced differentially expressed miRNAs in the line 6_3_ birds were significantly enriched (Bonferroni corrected *p* < 0.01) in a total of 237 GO terms, which included 17 molecular function (GO:MF), 172 biological process (GO:BP), and 48 cellular component (GO:CC) terms (Additional file 6; Figure 4), in addition to reactomes (REAC), WiKiPathways (WP), and transcription factors (TF). Most of the MF terms present binding functions, including protein binding, nucleic acid binding, organic cyclic compound binding, heterocyclic compound binding, DNA binding, RNA binding, enzyme binding, ion binding, and sequence-specific DNA binding. The BP terms encompassed cellular process, regulation of cellular and biological processes, regulation of gene expression, cell differentiation, cellular response to stimulus, signaling, signal transduction, and regulation of signal transduction. The CC terms are involved in membrane functionalities including membrane-bounded organelle, intracellular membrane-bounded organelle, membrane-enclosed lumen, membrane part, plasma membrane, intrinsic component of membrane, integral component of membrane, and plasma membrane bounded cell projection.

The target genes of CVI988/Rispens-induced differentially expressed miRNAs in the line 6_3_ birds were significantly over-enriched (Bonferroni corrected *p* < 0.01) in a total of 1057 GO terms, which included 118 GO:MF, 811 GO:BP, and 128 GO:CC terms (Figure 4). MF terms are heavily presented in binding functions including protein binding, ion binding, organic cyclic and heterocyclic compound binding, enzyme binding, anion binding, DNA binding, transcription regulatory region DNA binding, cation binding, small molecule binding, kinase binding, and ATP binding. MF terms are also broadly involved in biological activities including phosphoric ester hydrolase activity, transcription coregulator activity, channel activity, phosphatase activity, protein tyrosine phosphatase activity, ubiquitin protein ligase activity, enzyme activator activity, molecular transducer activity, and signaling receptor activity. BP terms are presented in cell adhesion, communication, cell cycle, cycle phase transition, cycle process, cell death, development, growth, and cell junction assembly and organization, cell migration and motility, cell–cell adhesion, signaling, and signaling by Wnt. The CC terms are involved in centrosome, chromatin, cytoplasm, dendritic tree, endomembrane system, intracellular organelle, organelle lumen, and intracellular vesicle. KEGG pathways including MAPK signaling pathway, Wnt signaling pathway, Hedgehog signaling pathway, and mTOR signaling pathway (Additional file 7).

Target genes of differentially expressed miRNAs between line 6_3_ and line 7_2_ birds in response to HVT vaccination were also heavily enriched in (221) MF terms, (1528) BP terms, and (250) CC terms. MF terms included variety of binding functions, such as ankyrin binding, ATP binding, ATPase binding, GTP binding, cytokine receptor binding, mRNA binding, myosin binding, and nuclear hormone receptor binding. MF terms also cover pyrophosphatase activity, Ras guanyl-nucleotide exchange factor activity, signaling activity, and signaling receptor activity. The BP terms involved in a variety of systems and functions including angiogenesis, apoptotic process, autophagy, biological adhesion, regulation and process, cell differentiation and division, cell-substrate adhesion and junction assembly, and cellular response to varied compounds and stimulus. The CC terms are involved in functions including cell surface, coated membrane, vesicle, vesical membrane, transcription factor complex, and whole membrane. In addition, the target genes were also enriched in KEGG pathways including adipocytokine signaling pathway, calcium signaling pathway, C-type lectin receptor signaling pathway, ErbB signaling pathway, FoxO signaling pathway, mRNA surveillance pathway, mTOR signaling pathway, and TFG-beta signaling pathway (Additional file 8). Additional GO terms and pathways for the target genes from comparisons between CVI988/Rispens and HVT in lines 6_3_ and 7_2_, between lines 6_3_ and 7_2_ in response to CVI988/Rispens, and line 7_2_ birds in response to CVI988/Rispens are given in rest of the Additional files 9, 10, 11, 12.

## Discussion

Marek’s disease has been well under controlled since the 1970s, which, in large, is attributable to the wide use of MD vaccines in poultry flocks [9]. Commonly used commercial MD vaccines include the first-generation anti-virus-induced tumor vaccine HVT, then the second comer SB-1, and the current gold-standard MD vaccine CVI988/Rispens [39, 40]. While most researchers and industry professionals, if not all, fully recognize the great good that MD vaccines have done for the poultry industry, few, if any, claim how the MD vaccines protect chickens against the MDV-induced tumors is thoroughly understood, including immunologists [41]. The reality that this paradox persistently remains bars the advancement of knowledge-based new vaccine design and development.

Genetic mechanism underlying how MD vaccine inserts protection against MD tumor formation is also far from fully understood. Earlier studies demonstrated that *MHC* plays an important role in modulating MD vaccine protective efficacy in chicken [21–23, 42, 43]. Our recent studies using the highly inbred lines of chickens, lines 6_3_ and 7_2_ carrying the same MHC B*2 haplotype, showed that non-*MHC* genomic contents also play a significant role in enabling chicken’s ability to convey protection against MDV-induced tumor formation in response to MD vaccines. Our experimental data clearly showed the line 6_3_ birds convey a very high protection rate, which strikingly differs from the line 7_2_ birds in response to HVT. The CVI988/Rispens delivers similar protection to the line 6_3_ but a little better protection to line 7_2_ than HVT [25, 28].

Epigenetics has been demonstrated to play an important role in orchestrating key biological processes, which are implemented through a number of factors including DNA methylation, histone modification, and ncRNAs [44, 45]. The epigenetic factors, in turn, are continuously modified throughout life in response to environmental exposures [45, 46]. Epigenetics refers to the study of changes in gene expression that occur without a change in DNA sequence. DNA methylation involves the addition of a methyl group to the carbon-5 of the cytosine pyrimidine ring and typically occurs at CpG sites containing cytosine-guanine nucleotides in a linear sequence. CpG islands, short stretches of DNA with rich CpG sites, are often found at promoters of mammalian genes. DNA methylation at these sites is highly correlated with transcription status of corresponding genes. Histone modification defines discrete chromatin regions with distinct structures associated with distinct transcription states of genes. miRNAs are small-non-coding RNAs. Mature miRNAs are ~23 nucleotides in length and regulate gene expression by preventing the translation of specific mRNAs. Epigenetic mechanisms are multifaceted and complex, which provide an additional layer of transcriptional control and a layer of post-transcriptional control to regulate gene expression, and, consequently, gene function [47–52]. A very recent study using small RNA_Seq identified 24 cellular miRNAs with altered expression in response to HVT, MDV, or both inoculations. The report claims cellular miRNAs are of critical players both in protection against and mediating progression of MD [53].

Up to date, there are 2114 unique gga-miRNAs that have been coined with accession numbers (miRbase release 22.1: October 2018). This study identified a total of 693 miRNAs including 198 reported gga-miRNAs and 495 novel miRNAs in the line 6_3_ and 7_2_ chickens. The identified novelMiRs were over twice as many as the identified known gga-miRNAs in bursae of the line 6_3_ and 7_2_ chickens 26 days post MD vaccine inoculation (Additional file 1).

Notable difference in the number of differentially expressed miRNAs were observed in the line 6_3_ birds. Vaccine and challenge trials repeatedly showed that HVT provides equally well or even better protection against MD in response to very virulent plus MDV challenge [25, 28], yet only four differentially expressed miRNAs were identified in line 6_3_ in response to HVT inoculation, but 13 differentially expressed miRNAs were identified in response to CVI988/Rispens in this line. Further, a direct comparison between CVI988/Rispens and HVT inoculation of line 6_3_ birds resulted in a total of 28 differentially expressed miRNAs. All these differentially expressed miRNAs (Table 1) might in different ways at different levels through their target genes as well as the GO terms and pathways insert influence to mediate each vaccine’s protective efficacy against MD.

In contrast, direct comparisons between the lines 6_3_ and 7_2_ birds 26 days post-vaccination resulted in a total of 34 and 30 differentially expressed miRNAs in response to HVT and CVI988/Rispens inoculation, respectively (Table 3). It is interesting to note that the four differentially expressed miRNAs identified between line 6_3_ HVT and line 6_3_ control groups (Table 1) were also among the 34 differentially expressed miRNAs of the line 6_3_ and 7_2_ comparison inoculated with HVT. Not only so, the log_2_ FC positive and negative signs of the four differentially miRNAs were also consistent, and the magnitude of fold change were similar. Knowing that HVT does not induce much protection to line 7_2_ but great protection for line 6_3_ birds, it is speculated here that the additional 30 differentially expressed miRNAs identified between lines 6_3_ and 7_2_ post HVT inoculation might be also important in modulating HVT protective efficacy in chickens like the line 6_3_ birds.

In summary, a relatively large number of differentially expressed microRNAs were identified in two highly inbred lines of chicken post MD vaccine inoculation. These two genetic lines of chickens differ in the capacity to convey protective efficacy against MDV-induced tumor formation in response to MD vaccination based on previous studies. Therefore, this finding may serve as the first piece of experimental evidence that the epigenetic factor, microRNA, is highly likely involved in modulating vaccine protective efficacy in chicken through their target genes in combination with varied biological processes and pathways.

## Supplementary information

**Additional file 1: Complete list of unique microRNAs identified in all biosamples of this study.** Unique microRNAs including known microRNAs and novel microRNAs identified in two lines of chickens in response to vaccination.

**Additional file 2: miRNAs identified in bursae of Line 6**_**3**_**chickens 26 days post HVT inoculation.** A microRNA profile of line 6_3_ in response to HVT vaccination.

**Additional file 3: miRNAs identified in bursae of Line 6**_**3**_**chickens 26 days post Rispens inoculation.** A microRNA profile of line 6_3_ in response to CVI988/Rispens vaccination.

**Additional file 4: miRNAs identified in bursae of Line 7**_**2**_**chickens 26 days post HVT inoculation.** A microRNA profile of line 7_2_ in response to HVT vaccination.

**Additional file 5: miRNAs identified in bursae of Line 7**_**2**_**chickens 26 days post Rispens inoculation.** A microRNA profile of line 7_2_ in response to CVI988/Rispens vaccination.

**Additional file 6: GO terms enriched by target genes of differentially expressed miRNAs in line 6**_**3**_**bursae 26 days post HVT inoculation.** Gene Ontology analysis of a list of target genes of differentially expressed microRNAs of the line 6_3_ birds in response to HVT vaccination.

**Additional file 7: GO terms enriched by target genes of differentially expressed miRNAs in line 6**_**3**_**bursae 26 days post Rispens inoculation.** Gene Ontology analysis of a list of target genes of differentially expressed microRNAs of the line 6_3_ birds in response to CVI988/Rispens vaccination.

**Additional file 8: GO terms enriched by target genes of differentially expressed miRNAs in bursae between line 6**_**3**_**and line 7**_**2**_**birds 26 days post HVT inoculation.** Gene Ontology analysis of a list of target genes of differentially expressed microRNAs between line 6_3_ and line 7_2_ birds 26 days post HVT vaccination.

**Additional file 9: GO terms enriched by target genes of differentially expressed miRNAs in bursae between Rispens and HVT vaccinated line 6**_**3**_**birds 26 days post-inoculation.** Gene Ontology analysis of a list of target genes of differentially expressed microRNAs between Rispens and HVT inoculated line 6_3_ birds 26 days post-vaccination.

**Additional file 10: GO terms enriched by target genes of differentially expressed miRNAs in bursae between Rispens and HVT vaccinated line 7**_**2**_**birds 26 days post-inoculation.** Gene Ontology analysis of a list of target genes of differentially expressed microRNAs between Rispens and HVT vaccinated line 7_2_ birds 26 days post-vaccination.

**Additional file 11: GO terms enriched by target genes of differentially expressed miRNAs in bursae between line 6**_**3**_**and line 7**_**2**_**birds 26 days post Rispens inoculation**. Gene Ontology analysis of a list of target genes of differentially expressed microRNAs between line 6_3_ and line 7_2_ birds 26 days post CVI988/Rispens vaccination.

**Additional file 12: GO terms enriched by target genes of a differentially expressed miRNA in bursae of line 7**_**2**_**birds in response to Rispens vaccination.** Gene Ontology analysis of a list of target genes of a differentially expressed microRNA of line 7_2_ birds 26 days post CVI988/Rispens vaccination.

## References

[1] Marek J (1907). Multiple Nervenentzündung (Polyneuritis) bei Hühnern. Dtsch Tierärztl Wochenschr.

[2] Gimeno IM, Witter RL, Reed WM (1999). Four distinct neurologic syndromes in Marek’s disease: effect of viral strain and pathotype. Avian Dis.

[3] Xie QM, Chang S, Dong KZ, Dunn JR, Song JZ, Zhang HM (2017). Genomic variation between genetic lines of White Leghorns differed in resistance to Marek’s disease. J Clin Epigenet.

[4] Niu S, Jahejo AR, Jia FJ, Li X, Ning GB, Zhang D, Ma HL, Hao WF, Gao WW, Zhao YJ, Gao SM, Li GL, Li JH, Yan F, Gao RK, Bi YH, Han LX, Gao GF, Tian WX (2018). Transcripts of antibacterial peptides in chicken erythrocytes infected with Marek’s disease virus. BMC Vet Res.

[5] Calnek BW (2001). Pathogenesis of Marek’s disease virus infection. Curr Top Microbiol Immunol.

[6] Brochu NM, Guerin MT, Varga C, Lillie BN, Brash ML, Susta L (2019). A two-year prospective study of small poultry flocks in Ontario, Canada, part 2: causes of morbidity and mortality. J Vet Diagn Invest.

[7] Gimeno IM (2008). Marek’s disease vaccines: a solution for today but a worry for tomorrow?. Vaccine.

[8] Rozins C, Day T, Greenhalgh S (2019). Managing Marek’s disease in the egg industry. Epidemics.

[9] Biggs PM, Nair V (2012). The long view: 40 years of Marek’s disease research and Avian Pathology. Avian Pathol.

[10] Smith J, Sadeyen JR, Paton IR, Hocking PM, Salmon N, Fife M, Nair V, Burt DW, Kaiser P (2011). Systems analysis of immune responses in Marek’s disease virus-infected chickens identifies a gene involved in susceptibility and highlights a possible novel pathogenicity mechanism. J Virol.

[11] Schat KA, Calnek BW (1978). Protection against Marek’s disease-derived tumor transplants by the nononcogenic SB-1 strain of Marek’s disease virus. Infect Immun.

[12] Rispens BH, van Volten H, Mastenbroek N, Maas JL, Schat KA (1972). Control of Marek’s disease in the Netherlands. II. Field trials on vaccination with an avirulent strain (CVI 988) of Marek’s disease virus. Avian Dis.

[13] Witter RL, Offenbecker L (1978). Duration of vaccinal immunity against Marek’s disease. Avian Dis.

[14] Baigent SJ, Smith LP, Nair VK, Currie RJ (2006). Vaccinal control of Marek’s disease: current challenges, and future strategies to maximize protection. Vet Immunol Immunopathol.

[15] Wozniakowski G, Niczyporuk JS (2015). Detection of specific UL49 sequences of Marek’s disease virus CVI988/Rispens strain using loop-mediated isothermal amplification. J Virol Methods.

[16] Baigent SJ, Nair VK, Le Galludec H (2016). Real-time PCR for differential quantification of CVI988 vaccine virus and virulent strains of Marek’s disease virus. J Virol Methods.

[17] Liu S, Sun W, Huang X, Zhang W, Jia C, Luo J, Shen Y, El-Ashram S, He C (2017). A promising recombinant herpesvirus of turkeys vaccine expressing PmpD-N of *Chlamydia psittaci* based on elongation factor-1 alpha promoter. Front Vet Sci.

[18] McKimm-Breschkin JL, Faragher JT, Withell J, Forsyth WM (1990). Isolation of very virulent Marek’s disease viruses from vaccinated chickens in Australia. Aust Vet J.

[19] Sharma JM, Witter RL (1983). Embryo vaccination against Marek’s disease with serotypes 1, 2 and 3 vaccines administered singly or in combination. Avian Dis.

[20] Spencer JL, Robertson A (1975). Influence of vaccination with avirulent herpesvirus on subsequent infection of chickens with virulent Marek’s disease herpesvirus. Am J Vet Res.

[21] Bacon LD, Hunt HD, Cheng HH (2000). A review of the development of chicken lines to resolve genes determining resistance to diseases. Poult Sci.

[22] Bacon LD, Witter RL (1994). Serotype specificity of B-haplotype influence on the relative efficacy of Marek’s disease vaccines. Avian Dis.

[23] Bacon LD, Witter RL (1994). B haplotype influence on the relative efficacy of Marek’s disease vaccines in commercial chickens. Poult Sci.

[24] Witter RL (2001). Protective efficacy of Marek’s disease vaccines. Curr Top Microbiol Immunol.

[25] Chang S, Xie Q, Dunn JR, Ernst CW, Song J, Zhang HM (2014). Host genetic resistance to Marek’s disease sustains protective efficacy of herpesvirus of turkey in both experimental and commercial lines of chickens. Vaccine.

[26] Tomasi TB, Magner WJ, Khan AN (2006). Epigenetic regulation of immune escape genes in cancer. Cancer Immunol Immunother.

[27] Dougan G, Highfield P (1985). Molecular biology, the new genetics and vaccine development. Med Lab Sci.

[28] Chang S, Dunn JR, Heidari M, Lee LF, Song J, Ernst CW, Ding Z, Bacon LD, Zhang H (2010). Genetics and vaccine efficacy: host genetic variation affecting Marek’s disease vaccine efficacy in White Leghorn chickens. Poult Sci.

[29] Heidari M, Zhang HM (2019) PRJNA543524. NCBI. https://www.ncbi.nlm.nih.gov/sra

[30] An J, Lai J, Lehman ML, Nelson CC (2013). miRDeep*: an integrated application tool for miRNA identification from RNA sequencing data. Nucleic Acids Res.

[31] Kozomara A, Birgaoanu M, Griffiths-Jones S (2019). miRBase: from microRNA sequences to function. Nucleic Acids Res.

[32] Kozomara A (2019) miRBase. http://www.mirbase.org/ftp.shtml

[33] Lewis BP, Burge CB, Bartel DP (2005). Conserved seed pairing, often flanked by adenosines, indicates that thousands of human genes are microRNA targets. Cell.

[34] Balcells I, Cirera S, Busk PK (2011). Specific and sensitive quantitative RT-PCR of miRNAs with DNA primers. BMC Biotechnol.

[35] Anders S, Pyl PT, Huber W (2015). HTSeq–a Python framework to work with high-throughput sequencing data. Bioinformatics.

[36] g:Profiler (2019) ELIXIR. http://biit.cs.ut.ee/gprofiler/index.cgi

[37] Reimand J, Kull M, Peterson H, Hansen J, Vilo J (2007). g:Profiler–a web-based toolset for functional profiling of gene lists from large-scale experiments. Nucleic Acids Res.

[38] Zhang HM, Heidari M (2019) PRJNA543526. NCBI. https://www.ncbi.nlm.nih.gov/sra/PRJNA543526

[39] Garcia M (2017). Current and future vaccines and vaccination strategies against infectious laryngotracheitis (ILT) respiratory disease of poultry. Vet Microbiol.

[40] Reddy SM, Izumiya Y, Lupiani B (2017). Marek’s disease vaccines: current status, and strategies for improvement and development of vector vaccines. Vet Microbiol.

[41] Boodhoo N, Gurung A, Sharif S, Behboudi S (2016). Marek’s disease in chickens: a review with focus on immunology. Vet Res.

[42] Bacon LD, Witter RL (1992). Influence of turkey herpesvirus vaccination on the B-haplotype effect on Marek’s disease resistance in 15.B-congenic chickens. Avian Dis.

[43] Bacon LD, Witter RL (1993). Influence of B-haplotype on the relative efficacy of Marek’s disease vaccines of different serotypes. Avian Dis.

[44] Aslani S, Jafari N, Javan MR, Karami J, Ahmadi M, Jafarnejad M (2017). Epigenetic modifications and therapy in multiple sclerosis. Neuromol Med.

[45] van Otterdijk SD, Michels KB (2016). Transgenerational epigenetic inheritance in mammals: how good is the evidence?. FASEB J.

[46] Marsit CJ (2015). Influence of environmental exposure on human epigenetic regulation. J Exp Biol.

[47] Hamilton JP (2011). Epigenetics: principles and practice. Dig Dis.

[48] Ling C, Groop L (2009). Epigenetics: a molecular link between environmental factors and type 2 diabetes. Diabetes.

[49] Reddy MA, Natarajan R (2011). Epigenetic mechanisms in diabetic vascular complications. Cardiovasc Res.

[50] van Weerd JH, Koshiba-Takeuchi K, Kwon C, Takeuchi JK (2011). Epigenetic factors and cardiac development. Cardiovasc Res.

[51] Wulff BE, Nishikura K (2012). Modulation of microRNA expression and function by ADARs. Curr Top Microbiol Immunol.

[52] Yan C, Boyd DD (2006). Histone H3 acetylation and H3 K4 methylation define distinct chromatin regions permissive for transgene expression. Mol Cell Biol.

[53] Hicks JA, Liu HC (2019). Impact of HVT vaccination on splenic miRNA expression in Marek’s disease virus infections. Genes (Basel).

